# Identifying the Potential Mechanism of Action of SNPs Associated With Breast Cancer Susceptibility With GVITamIN

**DOI:** 10.3389/fbioe.2020.00798

**Published:** 2020-08-04

**Authors:** An-phi Nguyen, Paola Nicoletti, Damien Arnol, Andrea Califano, María Rodríguez Martínez

**Affiliations:** ^1^IBM Research–Zurich, Zurich, Switzerland; ^2^ETH-Zürich, Zurich, Switzerland; ^3^Herbert Irving Cancer Research Center, Columbia University Medical Center, New York, NY, United States; ^4^Department of Systems Biology, Columbia University, New York, NY, United States; ^5^Herbert Irving Comprehensive Cancer Center, Columbia University, New York, NY, United States; ^6^Department of Biomedical Informatics, Columbia University, New York, NY, United States; ^7^Department of Biochemistry and Molecular Biophysics, Columbia University, New York, NY, United States; ^8^Department of Medicine, Vagelos College of Physicians and Surgeons, Columbia University, New York, NY, United States; ^9^J.P. Sulzberger Columbia Genome Center, Columbia University, New York, NY, United States

**Keywords:** cancer-susceptibility SNP, SNP mechanism of action, transcription factor dysregulation, breast cancer, multi-omics integration, nonparametric hypothesis test, *p*-value combination

## Abstract

In the last decade, a large number of genome-wide association studies have uncovered many single-nucleotide polymorphisms (SNPs) that are associated with complex traits and confer susceptibility to diseases, such as cancer. However, so far only a few heritable traits with medium-to-high penetrance have been identified. The vast majority of the discovered variants only leads to disease in combination with other still unknown factors. Furthermore, while many studies aimed to link the effect of SNPs to changes in molecular phenotypes, the analysis has been often focused on testing associations between a single SNP and a transcript, hence disregarding the dysregulation of gene regulatory networks that has been shown to play an essential role in disease onset, notably in cancer. Here we take a systems biology approach and develop GVITamIN (Genetic VarIaTIoN functional analysis tool), a new statistical and computational approach to characterize the effect of a SNP on both genes and transcriptional regulatory programs. GVITamIN exploits a novel statistical approach to combine the usually small effect of disease-susceptibility SNPs, and reveals important potential oncogenic mechanisms, hence taking one step further in the direction of understanding the SNP mechanism of action. We apply GVITamIN on a breast cancer cohort and identify well-known cancer-related transcription factors, such as CTCF, LEF1, and FOXA1, as TFs dysregulated by breast cancer-associated SNPs. Furthermore, our results reveal that SNPs located on the RAD51B gene are significantly associated with an abnormal regulatory activity, suggesting a pivotal role for homologous recombination repair mechanisms in breast cancer.

## 1. Introduction

Complex diseases, such as cancer, are caused by the interaction of numerous genetic and environmental factors, most of them having a relatively small effect. *Single nucleotide polymorphisms* (SNPs) have been found to increase susceptibility to certain diseases including cancer. Genome wide association studies (GWAS) have sought to identify some of these associations between categorical phenotypes, e.g., cancer vs. non-cancer, and a limited number of common variants (Manolio, 2010). However despite intense efforts, only a few medium-to-high penetrance heritable traits have been identified, and the majority of heritable genetic risk factors for most common complex diseases remain elusive (Manolio et al., 2009). While SNPs might not be able to explain single-handedly cancer susceptibility, it has been suggested that GWAS data can yield additional insight when combined with other data modalities (Califano et al., 2012).

In parallel, statistical reconstruction of gene regulatory networks in healthy and diseased tissues has led to the identification of key transcription factors (TFs), named master regulators (Lefebvre et al., 2010), that are essential to the establishment and maintenance of a phenotype. Master regulators have been shown to be dysregulated in several human malignancies, such as prostate cancer (Aytes et al., 2014) and brain tumors (Carro et al., 2010). Similarly, prioritization of genes that are upstream of functional disease drivers has enabled the discovery of genetic alterations that are causal determinants of disease (Chen et al., 2014). Following a similar philosophy, we hypothesize here that a combined statistical analysis of the association between cancer susceptibility SNPs and dysregulated TFs might reveal important oncogenic mechanisms. Furthermore, GWAS typically reveal association signals, but rarely the causative events linking variants and phenotypes (Nicolae et al., 2010). Only in a few cases a SNP mechanism of action is known, as it is the case for instance, where a SNP changes a TF binding site (Kumar et al., 2017). However, recent studies suggest that only a minority of causal SNPs alter TF binding motifs (Farh et al., 2015), and indeed, it has been estimated that >90% of disease-associated SNPs lie outside protein coding regions, most likely affecting regulatory regions such enhancers (Hindorff et al., 2009; Ricaño-Ponce and Wijmenga, 2013) or non-coding RNAs (Hrdlickova et al., 2014). Therefore, an alternative promising approach to elucidate the way in which a SNP could contribute to disease is to analyse the dysregulatory molecular events induced by the SNP with the goal of shedding light on the biology of complex traits and etiological pathways.

In this work we seek to elucidate some of the potential mechanisms by which SNPs contribute to gene dysregulation in complex diseases. Specifically, we search whether a cancer-associated SNP alters gene expression levels and/or disrupts TF activity by testing the changes in transcriptional profiles conditioned on the presence of the SNP. Unlike standard expression quantitative trait loci (eQTL) analysis (Nica and Dermitzakis, 2013), which tests for direct associations between SNPs and gene expression changes, we perform a regulatory analysis, where we investigate not only direct changes in gene expression, but also changes in the regulatory activities of TFs. To that end, we introduce a novel statistical pipeline, GVITamIN (genetic VarIaTIoN functional analysis tool). GVITamIN performs two different types of analyses. In a first step, it tests whether a SNP is significantly associated with changes in gene expression. In the second step, it analyzes the correlation of TFs with their targets in order to uncover TFs whose regulatory activity is affected by the presence of a SNP. As a proof of concept, we focus on breast cancer, the most commonly occurring cancer in women and the second most common cancer overall. Previous GWAS have identified numerous SNPs that are associated with an increased risk of breast cancer, however the impact of these SNPs on molecular phenotypes has not been investigated in depth.

## 2. Materials and Methods

### 2.1. Overview of the Computational Approach

GVITamIN implements two different search strategies, a *first- and a second-order analysis*. In the *first-order analysis*, a differential gene expression analysis conditioned on the presence of the SNP is performed. Unlike eQTL analyses (Nica and Dermitzakis, 2013), which typically look for direct associations between a SNP and gene expression levels in a healthy cohort (Nica and Dermitzakis, 2013), here we ask whether there is an excess of cases who are either homozygotes or complex heterozygotes for a particular SNP in a *breast cancer cohort*. In doing so, we are distinguishing variants that confer a dominant vs. recessive effect on the risk of a disease, either as homozygotes or compound heterozygotes.

**Figure 1:**
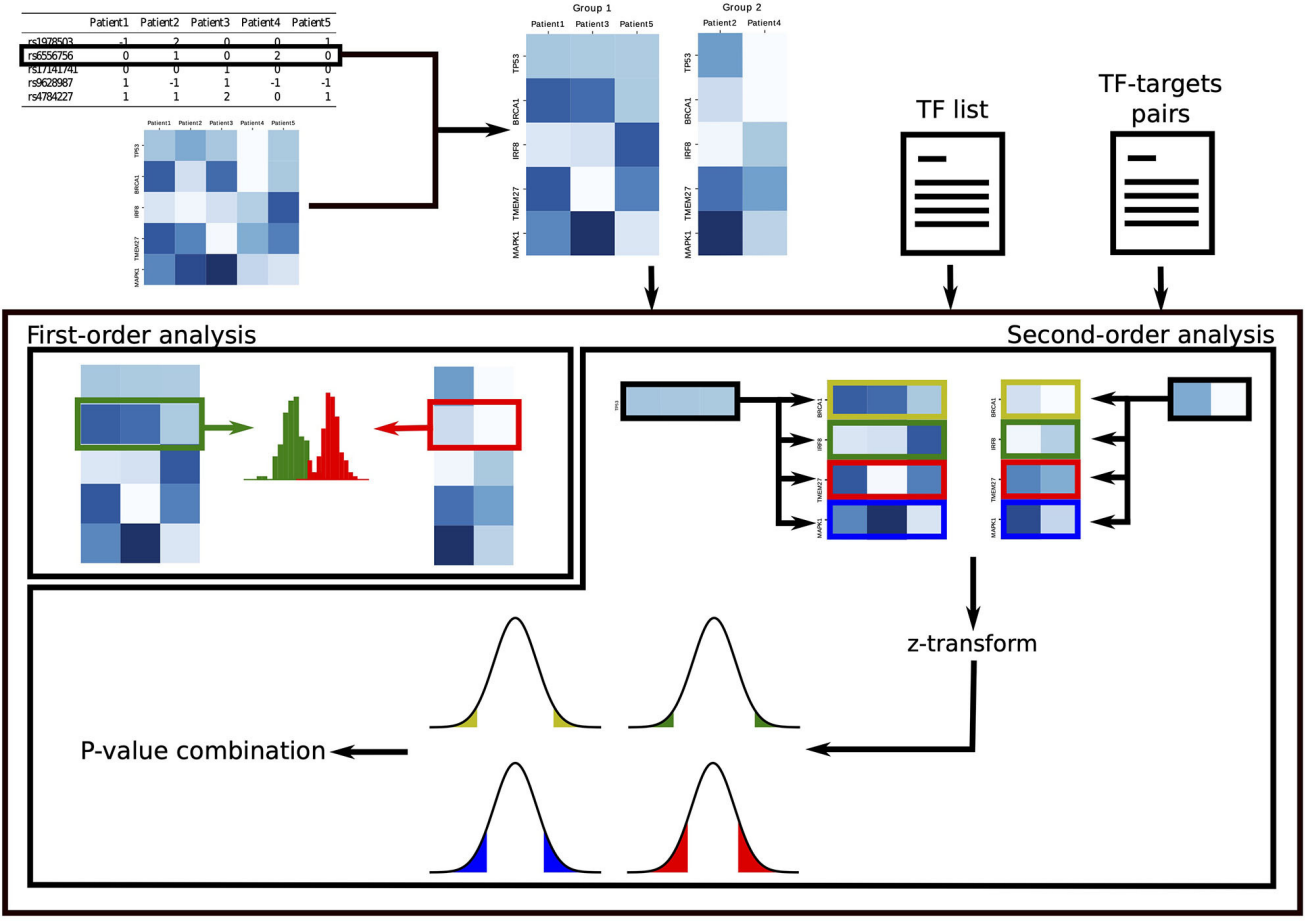
gVITaMIN pipeline. For the analysis on each SNP, we split the patients in two cohorts according to their allele. In the first-order analysis, we compare the distribution of gene expressions of the two cohorts using the non-parametric Mann-Whitney *U*-test. In the second-order analysis, given a list of Transcription Factors and a (optional) list of TF-TGs pairs, we compute correlations between TFs and their targets. After Fisher's z-transform, we test abnormal regulation activity for each TF-TG pair via a *z*-test. We finally test the global dysregulation of each TF by combining the *p*-values over its targets.

In the *second-order analysis*, we focus on discoverying SNPs that disrupt the regulatory activity of TFs, where disruption is measured as a change in correlation between the TF and its target genes. To uncover these events, we develop a novel statistical approach that exploits non-parametric statistical tests. To increase statistical power, the results of this analysis are combined into a single *p*-value for each TF, which quantifies the overall effect of the SNP on the TF and points toward dysregulated oncogenic programs driven by the SNP. Figure 1 summarizes GVITamIN computational approach, and additional details are given in subsections 2.2 and 2.3.

To perform both first- and second-order analyses, we separate the cohort in two groups according to the SNP genotyped allele. Namely, for each SNP, we split the cohort in three groups according to the sample's alleles, i.e., as homozygous major, heterozygous and homozygous minor. If the two homozygous groups have a sufficiently large number of samples determined by an user-defined threshold, we compare the homozygous major vs. the homozygous minor and discard the samples that are heterozygous. Here the idea is to preserve the original signal as pure as possible, e.g., not mixing samples which have both the putative risk allele and the wild-type allele. If the number of samples in the homozygous minor group is not sufficiently large to enable a significant study, we merge the heterozygous group with the lower count group. In this paper, we take *threshold*_*samples* = 30, although this value can be adapted to study cohorts of varying size. If, after merging, either of the two groups is smaller than *threshold*_*samples*, the SNP is discarded for subsequent analysis. We perform this analysis separately for each SNP.

It is easy to notice that the workflow can be easily parallelized on at least two levels. Firstly, it can be run in parallel on different SNPs. Since the analyses on different SNPs are independent, there is no need to aggregate the results in the end and, hence, there is no communication overhead. Moreover, for each SNP, the statistical tests can be performed in parallel for each gene or transcription factor. We implemented GVITamIN in C++, using the MPICH and OpenMP libraries to distribute the workloads. Code and data is available at https://ibm.box.com/s/e0u3gtp807ahikvilssg4717na24jpxt.

### 2.2. First-Order Analysis

#### 2.2.1. Detecting Differential Gene Expression

In the first-order analysis, we use the *Mann-Whitney U-test* (Mann and Whitney, 1947) to find SNPs that associate with the differential expression of some genes. The test is performed by computing a statistic U, which depends on the number and ranks of the gene expressions of the samples in each group. Note that the distribution of U under the null hypothesis is known. Further details can be found in the Supplementary Material.

#### 2.2.2. Multiple Hypotheses Correction

Sequencing technologies typically generate measurements of a large number of genes, in the order of tens of thousands. Simultaneously testing all these genes will lead to an inflated type I error (Dickhaus, 2014), i.e., a large number of false positives. This is commonly known as the *multiple comparisons problem* and is usually solved by correcting the *p*-values of the tests. Among the many ways to apply this correction, we implement a *False Discovery Rate* (FDR) controlling procedure designed by Benjamini and Yekutieli (2001) (BHY). Compared to other strategies, such as the ones controlling the *Family-Wise Type I Error* (FWER) (Dickhaus, 2014), FDR-based techniques have been shown to be less stringent and to have higher statistical power (Genovese, 2004).

### 2.3. Second-Order Analysis

This analysis is carried out in two levels. First, for each SNP, we test if the correlation between a TF and its targets genes (TGs) is perturbed. Second, to draw global conclusions, we combine the results obtained for the genes targeted by the same TF. Details about both computational analyses follow.

#### 2.3.1. Testing TF-TG Correlations

Using the SNP-associated groups previously defined (section 2.1), our aim is to test if the relationship of a TF-TG pair changes conditioned on the presence of the SNP. This can be done by comparing the correlations between TFs and TGs. Namely, we first compute the correlations between TGs and TFs ρ_*i*_ for each group *i* ∈ {1, 2}. We then transform the correlations into normally distributed variables using the Fisher's z-transform (Fisher, 1915):

(1) $$\begin{array}{l} z_{i}=\frac{1}{2}\ln\left(\frac{1+\rho_{i}}{1-\rho_{i}}\right)=\text{arctanh}(\rho_{i}). \end{array}$$

If the samples used to compute the correlations are independent and identically distributed, then *z* is approximately normally distributed with mean given by Equation (1) and variance:

(2) $$\begin{array}{l} \sigma_{i}^{2}=\frac{1}{N_{i}-3}, \end{array}$$

where *N*_*i*_ is the size of group *i*. Hence, correlations can be compared using a z-test with null hypothesis:

(3) $$\begin{array}{l} H_{0}:\tilde{z}=\frac{z_{1}-z_{2}}{\sqrt{\frac{1}{N_{1}-3}+\frac{1}{N_{2}-3}}}\sim N\left(0,1\right), \end{array}$$

where N(0,1) is the standard normal distribution. The method described above was derived for Pearson's correlation, but it was empirically shown to work with Spearman's correlation as well. In the latter case, Equation (2) becomes $\sigma_{i}^{2}=1.06/\left(N_{i}-3\right)$ (Fieller et al., 1957). Consequently, the *z* − *score* has to be corrected to

(4) $$\begin{array}{l} H_{0}:\tilde{z}=\frac{z_{1}-z_{2}}{\sqrt{\frac{1.06}{N_{1}-3}+\frac{1.06}{N_{2}-3}}}\sim N\left(0,1\right), \end{array}$$

GVITamIN can be run using either Pearson or Spearmann correlation. In addition, given the high number of tested TF-TG pairs, we again apply the BHY procedure to correct for multiple comparisons.

#### 2.3.2. Joint Analysis of TF Dysregulation

Once the correlation of individual TF-TGs pairs has been computed, we combine the correlations values obtained in each group by means of the *Fisher's method* (Fisher, 1992) to combine independent *p*-values:

(5) $$\begin{array}{l} X^{2}=-2\overset{T}{\underset{k}{\sum }}\ln(p_{k}), \end{array}$$

where *T* is the number of TGs of the analyzed TF. Under the null hypothesis, i.e., assuming that the *p*_*k*_ come from independent observations, *X*^2^ follows a χ^2^ distribution with 2*T* degrees of freedom.

The reasons for combining individual pairs predictions are 2-fold. First, from a biological point of view, we are usually interested in investigating the dysregulation of key TFs, which we expect to be associated with important oncogenic pathways. Second, from a statistical point of view, the signal associated with an individual TF-TG can be expected to be small, as SNPs typically only capture a small fraction of the genetic variability (Manolio et al., 2009). Aggregating the predictions associated with different TGs might therefore result in a stronger signal that would have been otherwise undetected after correcting for multiple testing.

#### 2.3.3. Brown Correction for Non-independent Tests

As mentioned before, the Fisher's method can only be applied to independent tests, which is usually not the case when analyzing large sets of correlated genes. The *Brown correction* (Brown, 1975) handles data dependencies by adjusting the combined test statistics with two rescaling factors, *c* and *f*, such that the rescaled distribution *cχ*^2^ follows a *X*^2^ distribution with *f* degrees of freedom. The factors *c* and *f* are computed from the correlation coefficients between the statistics of each test, i.e., the $\tilde{z}$ in Equation (3).

GVITamIN provides two methods to compute these coefficients. The first is based on an asymptotic closed formula that computes the correlations from various moments and cross-moments of the gene expression levels. We refer to the Supplementary Material for details on the derivation. Alternatively, it is possible to generate for each TF bootstrap estimates of the $\tilde{z}$ statistics by sampling the patients with replacement. The correlation coefficients to compute *c* and *f* are then obtained by applying Pearson's correlation formula on these bootstrap estimates. This second method is recommended for a relatively low number of genes and TFs. In this work, given the computational burden incurred in applying the second method, we applied only the asymptotic approximation.

Finally, after the Brown correction, we obtain a *combined p-value* for each transcription factor. Since we are simultaneously testing multiple TFs, we apply again the BHY procedure to control the FDR.

### 2.4. Input Data and Pre-processing

The required inputs to GVITamIN are a genotype matrix, a matrix of gene expression levels per sample and a list of transcription factors of interest. Both the genotype and gene expression levels are provided as matrices, where columns represent a single patient in the cohort and rows correspond, respectively to a SNP or a gene. Optionally, it is possible to provide an additional file containing a list of target genes for each transcription factor. If this file is not provided, the second-order analysis is performed for all possible TF-target gene pairs. Sample data is available for download at https://ibm.box.com/s/e0u3gtp807ahikvilssg4717na24jpxt.

#### 2.4.1. Gene Expression Matrix

We use level 3 RNAseq gene expression data from TCGA, which is already quality-controlled and normalized. Genes are filtered according to two criteria: (i) We limit our analysis to protein-coding genes to facilitate the interpretation of the results. (ii) To limit computational cost and excessive statistical power decrease due to testing too many hypotheses, we exclude uninformative genes, i.e., those genes with almost constant expression levels across our cohort. We note that if the first- and second-order analyses are run using Spearman's correlation, which is invariant under monotonic transformations, further common transformations, such as *log* or *arcsinh*, are unnecessary.

#### 2.4.2. SNP Genotype Matrix

The goal of GVITamIN is to unveil possible molecular mechanisms behind SNPs associated with complex diseases, such as breast cancer, and hence, we focus on the analysis of 59 SNPs already known to increase the risk of breast cancer. The list is obtained by overlapping the SNPs mentioned on SNPedia^1^ with the SNPs for which the data was available from TCGA. This list can be found in the Supplementary Material Section 2.

For the selected SNPs, we obtain a genotype matrix using TCGA low coverage DNA sequencing data and PLINK (Purcell et al., 2007), http://zzz.bwh.harvard.edu/plink/index.shtml. The entries of the matrix are integer numbers indicating for each SNP to which genotype group a patient belongs: 0, 1, and 2 stands, respectively for homozygous major allele, heterozygous and homozygous minor allele. A special value can be set by the user, e.g., “−1,” to indicate missing values or low-confidence calls that should be discarded for further analysis.

#### 2.4.3. Definition of TF-TGs Interactions

To limit the amount of tested hypothesis and increase GVITamIN statistical power, we limit our search to known TF-TG interactions that are supported by experimental evidence, excluding hence purely computational predictions. Specifically, we compile a list of TF-TG interactions using the following databases: ITFP (Zheng et al., 2008), which predicts TF and regulatory interactions from protein sequences and gene expression data using statistical models, and validates them based on experimental evidence from orthologous genes from other mammalian species; the ENCODE project (Consortium, 2012), which identifies TF binding sites (TFBS) using ChIP-seq data and infers the interacting target genes based on the distance of gene transcription starting sites (TSS) from the TFBS; the TRANSFAC project (Wingender, 2008), which follows a similar strategy, while leveraging experimental evidence from different technologies, such as DNase footprinting; TRRUST (Han et al., 2018), a manually curated database of regulatory interactions discovered by text-mining millions of publications. An additional resource provided by Marbach et al. (2016), which uses the cap analysis gene expression (CAGE) (Shiraki et al., 2003), produced a high number of interactions leading us to suspect a high false positive rate. Therefore, we discard this dataset from our lists. The final list of transcription factors and targets interactions is obtained by aggregating the four selected databases and further filtered as explained in the previous section. The list can be downloaded at https://ibm.box.com/s/e0u3gtp807ahikvilssg4717na24jpxt.

#### 2.4.4. Subsampling

To increase the robustness of our results, we re-run GVITamIN on a partition of the original data containing 85% of the samples (without repetition). We managed to run GVITamIN on 272 such partitions in 2 days of running time. To filter out low confidence results, we only keep those results predicted in the *main run*, i.e. the run using all the samples, as well as in *all* the 272 subsampling runs. The goal of this procedure is to identify effects which are strong enough to be detected using only subsets of the samples. With this approach, we are reducing the false positive rate at the cost of a slight increased of the false negative rate.

#### 2.4.5. Computing Infrastructure

We run our pipeline using 15 MPI processes and 16 OpenMP threads per MPI process on a cluster equipped with POWER8 processors. In such a setting our code run in about 34 min.

## 3. Results

We test GVITamIN on a cohort of breast cancer patients collected by the TCGA Research Network^2^. After the pre-processing described in section 2.4, we obtain expression levels for 15,669 genes and 1,060 patients, which we use to investigate the molecular dysregulation caused by 59 *breast cancer-related* SNPs. For the second-order analysis, we focus on 766 TF and 114,637 TF-targets interactions as described in 2.4.

### 3.1. First Order Results

In the first order analysis, we test the association of 59 SNPs with the changes in expression levels of 15669 genes. GVITamIN rejected 17925 out of the 924471 tests at the 0.05 significance level (after FDR correction). After running GVITamIN on various subsamples of the data (section 2.4), we select 3231 rejected tests out of 17925 initial results for further analysis.

#### 3.1.1. Top Results

We report in Table 1 the top 10 results ranked in terms of the FDR-corrected *p*-value. Most of these top results are related to 2 SNPs, rs4455437 and rs2842347.

**Table 1:** First order analysis: top 10 most significant first order results.

**SNP**	**Gene**	***p*-value**	**FDR *p*-value**
rs4455437	LCE1F	1.09224e-80	1.75194e-75
rs4455437	LCE2D	8.99746e-80	7.21591e-75
rs4455437	LCE2C	7.94305e-79	4.24685e-74
rs4455437	LCE1A	5.00239e-78	2.00594e-73
rs2842347	KRTAP9-4	1.20898e-77	1.93918e-72
rs2842347	GAGE12J	1.00726e-76	8.07816e-72
rs4455437	LCE1E	7.45837e-75	2.39262e-70
rs2842347	SPRR2B	2.71334e-74	1.45072e-69
rs2842347	KRTAP4-12	1.34211e-73	5.38182e-69
rs7107217	LCE6A	2.14795e-72	3.44529e-67

*Most of the top results involve genes from the late cornified envelope (LCE) family and SNP rs445543. This SNP is located close to the gene TNIP3, which has been linked to breast cancer in African American women*.

rs4455437 is a SNP located ~30 kb downstream of the TNIP3 gene and was recently associated with breast cancer in African American women (Song et al., 2013). From our analysis, rs4455437 seems mostly associated with the genes of the late cornified envelope (LCE) family. Indeed, 11 genes of the 52 genes significantly associated with rs4455437 belong to the LCE gene family—LCE1A, LCE1B, LCE1C, LCE1D, LCE1E, LCE1F, LCE2A, LCE2B, LCE2C, LCE2D, LCE3E, LCE6A. We do not find any evidence of a differential expression of TNIP3 or of other cancer-related genes in the presence of rs4455437. Regarding rs2842347, it has also been recently associated with breast cancer in African American women in the same study cited above (Song et al., 2013). This SNP is located on chromosome 14 on the RAD51B gene, a known cancer gene involved in homologous recombination repair (HRR). Among the genes differentially expressed in the presence of rs2842347, we find genes involved in keratinization, including members of the LCE family.

#### 3.1.2. Gene Set Enrichment Analysis

We further analyze the first order results by performing an enrichment analysis (in the form of a *chi-square* test) against the following collections of gene sets from the Molecular Signatures Database (MSigDB) (Subramanian et al., 2005; Liberzon et al., 2011): (i) **Canonical pathways** describing biological processes and manually curated by domain experts. These pathways are derived from the BioCarta (Nishimura, 2001), KEGG (Kanehisa and Goto, 2000; Kanehisa et al., 2016, 2017), and the REACTOME (Fabregat et al., 2018) databases; (ii) **Cancer modules** (Segal et al., 2004), which includes additional gene sets compiled from other databases, such as KEGG and GO; (iii) **Oncogenic signatures**, includes additional pathways identified as often disregulated in cancer by analyzing microarray data; (iv) **Hallmark**, gene sets summarizing all the other databases provided in the MSigDB in 50 specific biological processes with minimal gene overlap.

Most of the SNPs studied in the first order analysis were associated with a relatively low number of differentially expressed genes. We therefore focus our analysis on 3 SNPs associated with more than 100 genes: rs421379, rs3784099, and rs2048672 (associated respectively with 1,329, 1,009, and 117 genes). Results for rs421379 and rs2048672 are reported in Table 2. We do not observe any significant result for rs3784099 after FDR correction.

**Table 2:** Gene enrichment analysis for the 2 of the SNPs associated with the highest number of genes: rs421379 and rs2048672.

**SNP (# DE genes)**	**Pathway**	***p*-value**	**FDR *p*-value**
rs421379 (1329)	TBK1.DF_DN	2.341e-07	0.005
	GCM_RAB10	2.489e-06	0.025
rs2048672 (117)	MODULE_54 (Cell cycle)	2.684e-18	0.005
	HALLMARK_G2M_CHECKPOINT	1.900e-06	0.013
	HALLMARK_E2F_TARGETS	1.990e-06	0.013
	REACTOME_CELL_CYCLE	4.897e-06	0.024

*rs421379 might be related to dysregulation of cell proliferation and apoptosis via TBK1. rs2048672 seems to play a role in the G2-M cell cycle checkpoint*.

For rs421379, we obtain two significantly enriched gene sets. *TBK1.DF_DN* (Barbie et al., 2009) is a gene set composed of genes down-regulated as a combination of an over-expressed oncogenic form of KRAS and the suppression of TBK1, a kinase that regulates cell proliferation, apoptosis, autophagy, and anti-tumor immunity (Helgason et al., 2013; Durand et al., 2018). *GCM_RAB10* includes genes located in the neighborhood of the RAB10 gene, a member of the RAS oncogene family. While KRAS and RAB10 belong to functionally different gene families, they are both part of the *Ras protein superfamily*, whose members function as monomeric G proteins that act as binary molecular switches that can regulate cell proliferation. Overactivation of Ras signaling can lead to cancer (Wennerberg et al., 2005). Interestingly, rs421379 is located on the Chromosome 5 relatively close (556 kb upstream of) to the ARRDC3 gene, which could play a role in the regulation of *G protein-coupled receptors* (Nabhan et al., 2010; Han et al., 2013).

In the presence of rs2048672, differentially expressed genes show an enrichment in gene sets related to *cell cycle*. Namely, pathways *HALLMARK _ E2F _ TARGETS* and *HALLMARK _ G2M _CHECKPOINT* suggest an association of rs2048672 with specific phases of cell cycle. During the G2-M checkpoint, cells are checked for defective DNA, and damage are repaired if necessary, before initiating mitosis. E2F transcription factors have been shown to play an important role in the regulation of genes involved in the G1-S phase of the cell cycle (Dyson, 1998; Nevins, 1998). However, more recent studies have shown evidence of some involvement of E2F proteins in the G2-M stage as well (Polager et al., 2002; Ren et al., 2002). In particular, Zhu et al. (2004) suggested that E2F are directly regulating the expression of mitotic genes.

### 3.2. Results of the Second Order Analysis

In the second order analysis we study the dysregulation of the interactions between 766 transcription factors and their target genes, for a total of 114,637 TF-targets interactions, conditioned on the presence of the 59 SNPs. We obtain 36 significant results at the 0.05 significance level after FDR correction. Given the low number of significant results we do not prioritize them by subsampling and we do not perform an enrichment analysis. We show the top 15 results in Table 3.

**Table 3:** Second order analysis: top 15 most significant results.

**SNP**	**TF**	**Target**	***p*-value**	**FDR *p*-value**
rs16882214	PDX1	INS	1.68881e-12	2.36652e-06
rs1876206	MYOD1	CRCT1	1.53497e-11	2.15094e-05
rs1876206	MYOD1	HTN1	9.40789e-11	6.59161e-05
rs10509373	HEY1	CDH23	3.46953e-10	0.000486182
rs1876206	MYOD1	CELA3A	2.07202e-08	0.00967838
rs1801270	SMARCA4	DGCR8	8.87854e-09	0.0124414
rs3784099	CDC5L	PCDHGC3	3.96714e-08	0.0185304
rs3784099	ATF1	CD2AP	2.97751e-08	0.0208618
rs1876206	MYOD1	NKX2-1	6.05328e-08	0.0212061
rs3784099	FOXA1	TM4SF1	2.0034e-08	0.0280735
rs10510102	CEBPB	MBNL3	2.283e-08	0.0319916
rs3784099	CTCF	SCAF8	5.86418e-07	0.035728
rs3784099	LMO2	LYL1	5.69159e-07	0.0362527
rs3784099	CTCF	TLK1	2.84675e-07	0.0362648
rs3784099	CTCF	C8G	3.36879e-07	0.0363128
rs3784099	CTCF	SFN	5.21694e-07	0.0365523

*Several of these TFs and targets have been implicated in cancer, such as PDX1, MYOD1, CTCF, LMO2, and INS*.

#### 3.2.1. Top Results

The most significant result involves the association of rs16882214 with the PDX1-INS pair. PDX1 is a TF of homeobox genes family important in differentiation and development of the pancreas, duodenum and antrum, which functions as a putative tumor suppressor in gastric cancer (Ma et al., 2008; Roy et al., 2016). In addition, polymorphisms in the INS have been reported to be associated with increased prostate cancer risk (Ho et al., 2003).

The second most significant result involves rs1876206, a SNP located on the FBN1 gene that is associated with the dysregulation of the TF MYOD1 and 4 of its targets (CRCT1, HTN1, CELA3A, and NKX2-1). Interestingly, recent studies suggest both a role for FBN1 (Wang et al., 2015) and MYOD1 (Cai et al., 2016) in breast cancer. Several of the TGs have been also implicated in cancer (Wu et al., 2016; Matsubara et al., 2017; Li et al., 2019).

Like rs2842347, identified in the first order analysis (Table 1), rs3784099 is located on the RAD51B gene. This SNP is involved in most of the significant second order results (27 out of 36). From Table 4 we note four transcription factors (CTCF, EP300, YY1, LMO2) whose correlation with *more than one* target is significantly perturbed in the presence of rs3784099. CTCF, EP300 and LMO2 are listed in the Catalog Of Somatic Mutations In Cancer (COSMIC) (Tate et al., 2018) as genes that are causally implicated in cancer if mutated. Also the multifunctional TF YY1 has been reported to have an oncogenic role (Zhang et al., 2011). In particular, in breast cancer, it seems to have a negative regulatory effect on p27, a cell cycle inhibitor protein (Wan et al., 2012). The target genes involved in these results are reported in Table 5. Among these genes, PICALM (targeted by EP300), DDX6 (targeted by YY1) and LYL1 (targeted by LMO2) are reported on the COSMIC cancer gene list.

**Table 4:** List of SNP-TF pairs ranked by number of significant targets.

**SNP**	**TF**	**# significant targets**
rs3784099	CTCF	5
rs1876206	MYOD1	4
rs3784099	EP300	4
rs3784099	YY1	4
rs3784099	LMO2	2
rs10509373	HEY1	1
rs10510102	CEBPB	1
rs16882214	PDX1	1
rs1801270	SMARCA4	1
rs3784099	ATF1	1
rs3784099	BDP1	1
rs3784099	CDC5L	1
rs3784099	ELF1	1
rs3784099	FOXA1	1
rs3784099	IRF2	1
rs3784099	JUND	1
rs3784099	MYOD1	1
rs3784099	SIN3A	1
rs3784099	SMC3	1
rs3784099	SOX9	1
rs3784099	SP3	1
rs4987047	RAD21	1

*Among the top pairs, CTCF, EP300, and LMO2 are reported on the COSMIC cancer gene list*.

**Table 5:** Significant targets for the top significant transcription factors with at least one significant target.

**SNP**	**TF**	**Significant targets**
rs3784099	CTCF	SCAF8, TLK1, C8G, SFN, RRP9
rs3784099	EP300	PICALM, RFXANK, MAD2L2, PMVK
rs1876206	MYOD1	CRCT1, HTN1, CELA3A, NKX-2
rs3784099	YY1	LYPLA2, ABCE1, WDR13, DDX6
rs3784099	LMO2	LYL1, CDH5

*Most TFs in this table have been associated with cancer. Interestingly, also several of the target genes are reported on the COSMIC cancer gene list, for example, PICALM (targeted by EP300), DDX6 (targeted by YY1), and LYL1 (targeted by LMO2)*.

### 3.3. Results of TF-Combined Second Order Analysis

Next, we combine the *p*-values obtained from the second order analysis, obtaining a single *p*-value for each TF and for each of the 59 SNPs. We reject 3,860 tests at the 0.05-significance level after FDR correction. It can be noted that this number is much higher compared to the second-order analysis on TF-target-SNPs triplets (section 3.2). This may be due to the fact that by combining *p*-values, we are effectively combining multiple evidences from different targets. Given the higher number of targets for each TF, the combined *p*-values may have higher chance to be significant. As for the first order analysis, we filter out minor results by running GVITamIN on subsamples of the data, finally retaining only 356 rejected tests. These tests involve 148 TFs, 48 of which can be found in the COSMIC cancer genes list. In Table 6 we report the top results of the TF-combined analysis, while in Table 7 we report the top 10 SNPs associated with the highest number of significant results.

**Table 6:** Results of the global second order analysis.

**SNP**	**TF**
rs2380205	LEF1
rs10822013	LEF1
rs421379	LEF1
rs3784099	EP300
rs3784099	LEF1
rs2842347	LEF1
rs11613298	FOXO4
rs12762549	LEF1
rs2842347	TCF3
rs2048672	LEF1
rs3784099	CTCF
rs10822013	TCF3
rs737387	MAZ
rs2842347	CTCF
rs3784099	YY1

*The table shows the top 15 most significant results. GVITamIN reported a zero p-value for all the association of the SNP-TF pairs. We therefore used a Chi-score to rank them. LEF1, which may play an important role in cancer invasion, migration and proliferation, is correlated with many SNPs*.

**Table 7:** Global second order analysis.

**SNP (# significant TFs)**	**Significant TFs**
rs3784099 (61)	AHR, **ATF1**, ATF2, **BCL11A**, **BCLAF1**, BDP1, BPTF (1.11e-16, 7.67e-15), CDC5L, CEBPB, **CHD2**, **CREB1**, **CTCF**, ELF1, ELF2, EN1, **EP300**, ETS1, **FOXA1**, **FOXO3**, GABPA, **HIF1A**, IRF2, JUND, **LEF1**, **LMO2**, MAFG, **MECOM**, MTF1, **MYB**, **NF1**, **NFE2L2**, NFYB, **PCBP1**, PGR, POU2F1, PRRX2, **RAD21**, **REL**, RFX5, RREB1, SETDB1, SIN3A, SIRT1 (5.93e-10, 2.90e-08), SIRT6 (8.22e-15, 5.10e-13), SMAD1, SMC3, SOX9, SP1, SP2, SP3, SPI1, STAT1, **SUZ12**, TAF1, **TCF12**, TCF4, TEAD1, TFDP1, USF2, YY1, ZNF143
rs10509373 (37)	**AR**, ATF2, **BCL11A**, **CREB1**, **CTCF**, EGR4, ELF1, ELF2, EN1, **EP300**, **ERG** (3.86e-10, 2.67e-08), **ESR1**, ETS1, ETS, **FOXA1**, FOXF2, FOXJ1, FOXL1 (1.44e-14, 1.21e-12), FOXM1, **FOXO1**, **HEY1**, IRF2, **MAF** (3.25e-13, 2.50e-11), **MAX**, MIF, NR3C1, **PBX1**, POU2F1, PRRX2, REST, RXRB, SP1, SP3, SREBF1, TCF4, YY1, **ZEB1**
rs2048672 (25)	**AR**, **BCL11A**, **CREB1**, E2F1, E2F6, EBF2, ELF2, ETS1, **FLI1** (9.85e-10, 7.67e-08), FOXM1, **GATA2**, **HMGA1**, KLF12 (6.77e-15, 7.34e-13), **LEF1**, **LMO2**, MIF, MSX1, NFYB, **PAX5**, **RAD21**, REPIN1, TEAD1, TFDP1, TGIF1, ZHX2
rs421379 (23)	**AR**, **BCL11A** (6.56e-06, 3.36e-04), CEBPB, **CTCF**, **DDIT3**, E2F1, **ESR1**, **FOXA1**, FOXC1, **GATA2**, **GATA3**, **HLF** (3.00e-15, 3.45e-13), **HMGA1**, **LEF1**, **LMO2**, MEF2C, **MYB**, **PPARG**, RUNX2 (1.11e-16, 1.36e-14), RXRB, STAT5A, **VHL** (1.16e-05, 5.72e-04), **ZEB1**
rs2842347 (21)	**AR**, **BCL11A**, **BCLAF1**, CEBPB, **CHD2**, **CTCF**, ELF2, **ESR1**, ESRRA, FOXC1 (2.22e-16, 3.07e-14), **LEF1**, PBX3, RFX5, SIN3A, TBP, **TCF3**, TEAD1, TRAF4, UBP1 (7.12e-14, 7.87e-12), USF2, **ZEB1**
rs4784227 (20)	**AR**, E2F1, EGR3, ELF1, **ESR1**, **FOXA1**, FOXC1, **FOXO1**, **GATA2**, HDAC2, IRF2, MAFG, **NF1**, NFKB1, RBL2 (2.57e-06, 1.78e-04), SOX5, TCF4, USF1, ZBTB7A, **ZEB1**
rs2380205 (18)	**AR**, CEBPD, **CTCF**, **DDIT3**, **EBF1**, **FOXO1**, **LEF1**, NR3C1, PBX3, POU2F1, REPIN1 (2.71e-04, 0.017), SMC3, SP1 (1.01e-05, 7.35e-04), TAF1, **TCF12**, TCF4, USF2, **ZEB1**
rs10822013 (18)	**CTCF**, E2F1, ELK1, **ESR1**, ESRRA, ETS2, **FOXA1**, **LEF1**, **MAX**, **MYB**, NR2F2, NR3C1, **PBX1** (2.38e-14, 3.28e-12), PGR, REST, RORA, **TCF3**, TGIF1
rs704010 (13)	BPTF, CDC5L (1.89e-15, 3.48e-13), **CTCF**, MAFF (1.15e-13, 1.82e-11), MAFK, **NF1**, POU2F1, POU3F2, **PPARG** (2.84e-12, 3.93e-10), SMARCC1 (1.03e-11, 1.26e-09), SMC3, SP2, UBP1
rs1314913 (10)	**BCL11A**, CEBPB, **EBF1** (2.40e-07, 2.37e-05), **FOXA1**, **GATA2** (1.21e-11, 1.59e-09), **GATA3** (1.89e-05, 1.51e-03), PAX2 (4.00e-4, 0.027), **RAD21**, **RB1** (1.11e-16, 1.71e-14), **ZEB1** (5.41e-05, 0.0042)

*We report the top 10 SNPs that are significantly associated with the highest number of TFs. The p-value and the FDR-corrected p-values are given in brackets for each TF. If no values are reported, it means that GVITamIN reported p-values of zero. We denote in bold characters cancer-related genes as reported in the COSMIC gene list*.

#### 3.3.1. Top Results

In Table 6, we observe that 7 out of the 15 top results involve the LEF1 transcription factor. A recent review article (Santiago et al., 2017) has highlighted the central role of LEF1 in cancer invasion, migration and proliferation, suggesting its use as a biomarker and potential target for treatments. While LEF1 is commonly seen as an important transcription factor in the Wnt/β-catenin signaling pathway, it can also function independently from it, with several possible downstream consequences. The fact that LEF1 was significantly associated with a relatively high number of SNPs, which are not in linkage disequilibrium, nor inducing the same cohort splitting (see Figure S1), in our analyses might be additional evidence in support of its pivotal role in breast cancer.

Almost all the results involve known cancer-related transcription factors reported in the COSMIC gene list. The only top results TFs not included in the list are MAZ and YY1. The connection of YY1 with cancer was already discussed in section 3.2. Regarding MAZ, a TF highly upregulated in chronic inflammatory disease and several human cancers, several studies have pointed its connection with breast cancer (Yu et al., 2017) and colon cancer (Triner et al., 2018).

#### 3.3.2. Top Transcription Factors

Motivated by the results involving LEF1, we study which TFs are significantly associated with the highest number of SNPs. Such analysis could reveal TFs frequently dysregulated by cancer-related SNPs and that are suspected hence of having an oncogenic role. We report in Table 8 14 TFs associated with 5 or more SNPs. Similarly to LEF1, these TFs are also associated with SNPs that are *not* in linkage disequilibrium *nor* inducing the same cohort splits.

**Table 8:** TFs significantly associated with the highest number of SNPs.

**TF**	**# associated SNPs**
FOXA1	10
CTCF	9
ESR1	8
AR	7
LEF1	7
ZEB1	7
BCL11A	6
E2F1	6
ELF2	6
POU2F1	6
CEBPB	5
GATA2	5
MYB	5
SP1	5

*Among these transcription factors, FOXA1, CTCF, ESR1, AR, LEF1, ZEB1, BCL11A, GATA2, MYB are established cancer-related genes*.

Among these TFs, 9 (FOXA1, CTCF, ESR1, AR, LEF1, ZEB1, BCL11A, GATA2, MYB) are well-known cancer-related genes. For instance, FOXA1 and ESR1 (also known as ERα) are part of a transcriptional network responsible of the control of gene expression patterns of *luminal A breast cancer* (Nakshatri and Badve, 2009). This is the most common breast cancer subtype (Fallahpour et al., 2017) and is characterized by responsiveness to hormonal therapies and, consequently, good prognosis (Nakshatri and Badve, 2009). Indeed, FOXA1 has been hypothesized to be a mediator of hormonal response in breast and prostate cancer (Robinson and Carroll, 2012), and a hormonal regulatory complex involving FOXA1, GATA2 and AR has been shown to control gene expression in prostate cancer (Zhao et al., 2016). Other important associations are readily found in the literature: MYB has been connected with anomalies in the regulatory mechanisms involving ERα (Ramsay and Gonda, 2008). BCL11A is over-expressed and its genomic locus frequently amplified in triple-negative breast cancer gene, suggesting a tumorigenic role in these tumors (Khaled et al., 2015). ZEB1 has a pivotal role in tumor progression and metastasis and can underlie chemotherapeutic resistance in breast cancer (Zhang et al., 2015, 2018).

CTCF deserves a special mention. A multifunctional transcription factor, CTCF plays a role in many types of cancer, including breast cancer, via *different mechanisms* (Oh et al., 2017). For example, the CTCF-cohesin complex is involved in the formation of topologically associated domains (TADs) and chromatin loops (Ghirlando and Felsenfeld, 2016). Dysregulation of CTCF might lead to abnormal 3D DNA structure impacting the normal functioning of the cell (Pinoli et al., 2020).

#### 3.3.3. Top SNPs

Finally, we focus on the SNPs that dysregulate a higher number of TFs. Table 7 reports 10 SNPs that are significantly associated with the dysregulation of at least 10 TFs. The TFs are reported on the right column.

rs3784099 is the SNP associated by a large margin with the highest number of TFs. This SNP, already identified in the first order analysis (section 3.1), is located on the RAD51B gene, an important paralog of RAD51 involved in homologous recombination repair (HRR) after double strand breaks. Most of double strand breaks occurring during the G2 phase of the cell cycle are repaired by HRR, which although less error-prone than the alternative non-homologous end-joining, it is far from error-free (Malkova and Haber, 2012; Cannan and Pederson, 2016). Mutations on HRR genes can rapidly result in further damages that impair many downstream processes.

Interestingly, two other SNPs in Table 7, rs2842347 and rs1314913, are also located on RAD51B, which could mean that they play a role similar to rs3784099. rs10822013 is located on ZNF365 which also plays a role in HRR (Zhang et al., 2013). rs704010 is a mutation of the gene ZMIZ1, which has been shown to regulate the activity of various cancer-related transcription factors, including AR (Sharma et al., 2003) and P53 (Lee et al., 2007).

Unfortunately, the other SNPs in Table 7 are located on non-coding RNA genes or intergenic regions whose functions are still unknown/unclear, rendering difficult their functional interpretation.

## 4. Discussion

We present here GVITamIN, a novel statistical tool to extract insights about the potential mechanism of action of disease-susceptibility SNPs associated with complex diseases, such as cancer. GVITamIN searches for direct perturbations in genes as well as dysregulation of transcription factor programs conditioned on the presence of cancer-associated SNPs. From a methodological point of view, we provide a theoretically well-grounded approach to summarize multiple weak evidences of SNP-induced molecular perturbations into statistically robust predictions about the oncogenic function of the SNP. Some of our main findings are discussed below.

### 4.1. The Central Role of RAD51B and the Homologous Recombination Repair Mechanism

Our results show that multiple cancer-susceptibility SNPs, namely rs3784099, rs2842347, and rs1314913, are located on RAD51B, a gene whose protein is essential for DNA repair by homologous recombination. The specific mechanism of action of each SNP differs, varying from inducing the differential expression of several genes of the LCE family, associated with breast cancer in African American women (section 2.2), to inducing the malfunctioning of several transcription factors implicated in cancer, e.g., CTCF, EP300, YY1, LMO2 (section 3.2). This result is not surprising, as disruptions in DNA repair pathways predispose cells to accumulating DNA damage (Kelley et al., 2014). An interesting question is whether these SNPs are functionally similar, i.e., whether they elicit the molecular dysregulation of the same pathways. Table 7 shows that the TFs dysregulated by rs3784099, rs2842347, and rs1314913 are different. In addition, the 3 SNPs induce different cohort splits (see Figure S1), which seems to indicate that each SNP affects RAD51B by means of a different molecular mechanism.

### 4.2. First Order Analysis Identifies Critical Carcinogenic Pathways

We tested the association of 59 cancer-susceptibility SNPs with changes in expression levels *in a breast cancer cohort*. While most SNPs were associated with a low number of dysregulated genes, rs421379, rs3784099, and rs2048672 were significantly associated with more than 100 genes. A gene set enrichment analysis performed on the identified genes revealed associated with key oncogenic pathways, such as *TBK1.DF_DN* (Barbie et al., 2009), a gene set that signals the over-expression of an oncogenic form of KRAS and the suppression of TBK1, a kinase that regulates cell proliferation, apoptosis, autophagy, and anti-tumor immunity (Helgason et al., 2013; Durand et al., 2018). Similarly, members of the *GCM_RAB10* pathway, which includes genes located in the neighborhood of the RAB10 gene, a member of the RAS oncogene family (Wennerberg et al., 2005), are targeted by several of the studied SNPs. Also intriguing is the identification of pathways associated with cell cycle and check points, such as *HALLMARK_E2F_TARGETS* and *HALLMARK_G2M _CHECKPOINT*, whose dysregulation has a critical and well-known role in carcinogenesis.

### 4.3. Cancer-Susceptibility SNPs Tend to Dysregulate Cancer-Associated Transcription Factors

Ranking TFs according to the number of significantly associated cancer-susceptibility SNPs reveals many well-known cancer-related transcription factors (see Table 8). Among them, FOXA1 and ESR1 form part of a transcriptional network responsible of the control of gene expression patterns in luminal A breast cancer (Nakshatri and Badve, 2009), the most common breast cancer subtype. Our results suggest that the number of genetic variants affecting the activity of a transcription factor could be used as a proxy of the susceptibility to a related trait (in our case, breast cancer) and its severity. This conclusion aligns with the common variant-common disease hypothesis (Reich and Lander, 2001), which states that common disease-causing variants can be found in all human populations which manifest a given disease. Since highly penetrant mutations are relatively rare (Greaves, 2015), it is expected that a higher number of lower penetrance variants are needed for a deleterious effect to manifest, e.g., developing breast cancer.

### 4.4. Future Work

Our analyses demonstrate the validity of a combined statistical approach that exploits knowledge about transcriptional regulation within the cell. Further work is however necessary to investigate whether a combination of both types of analyses (first and second order analysis) can provide deeper insights on transcriptional mechanisms. Another possible improvement is the extension of our pipeline to other types of regulatory mechanisms, or to higher-order interactions. For example, the second-order analysis does not consider TF-TG interactions which are mediated by a co-factor. We observe, however, that such higher-order analyses would require more samples to achieve statistical significance.

## 5. Conclusion

We have presented here GVITamIN, a new statistical and computational approach to characterize the potential effect of a SNP on both genes and transcriptional regulatory programs. As demonstrated in this work, our novel statistical approach is able to combine the usually small effect of disease-susceptibility SNPs to reveal important oncogenic mechanisms, which were corroborated with published previous studies. An inherent problem to many statistical approaches to analyse high-throughput data is the high number of false positive results, even after FDR correction. While the only robust approach to reduce the number of false positives is to limit the number of tests and/or increase the sample size, workarounds can be designed to prioritize the results. In GVITamIN we implement a *p*-value cutoff based on subsampling/bootstrapping. Other approaches, such as ranking the results according to the FDR *p*-values and setting a very stringent *p*-value might unfairly penalize SNPs with very unbalanced sample sizes (as a result of an unbalanced cohort split), i.e., where one group size is close to the acceptance threshold (*threshold*_*samples*) resulting in predictions characterized by larger *p*-values.

We emphasize that GVITamIN is not limited to SNPs only, but could be used to characterize any categorical mutation or structural alteration with the potential of affecting gene expressions levels, such as short tandem repeats mutations, epigenetic changes, mutations altering topologically associated domains (TADs), etc.

As a final remark, we note that gVITaMIN can not infer the causal structure of such mechanisms, which require either longitudinal studies that follow a cohort through time or functional studies. In the absence of those, our rigorous statistical analysis might help identify candidate explanations for further experimental validation from one single time point observational data.

## Data Availability Statement

Publicly available transcriptomic data from TCGA breast cancer samples (available at https://portal.gdc.cancer.gov/) was analyzed in this study. Preprocessed data is available at https://ibm.box.com/s/e0u3gtp807ahikvilssg4717na24jpxt.

## Ethics Statement

Ethical review and approval was not required for the study on human participants in accordance with the local legislation and institutional requirements. The patients/participants provided their written informed consent to participate in this study.

## Author Contributions

AN implemented the code. PN contributed toward the interpretation of the results. DA performed data analysis. AC and MR conceived the study and analysis. AN and MR wrote the manuscript with input from all authors. All authors contributed to the article and approved the submitted version.

## Conflict of Interest

AC is the founder, equity holder, consultant, and director of DarwinHealth Inc., a company that has licensed some of the algorithms used in this manuscript from Columbia University. Columbia University is also an equity holder in DarwinHealth Inc. The remaining authors declare that the research was conducted in the absence of any commercial or financial relationships that could be construed as a potential conflict of interest.

## Footnotes

^1^https://www.snpedia.com/index.php/Breast_cancer

^2^http://cancergenome.nih.gov/
