# Supplementary material for: Identifying the Potential Mechanism of Action of SNPs Associated With Breast Cancer Susceptibility With GVITamIN
Source: Front Bioeng Biotechnol. 2020 Aug 4;8:798. doi: 10.3389/fbioe.2020.00798 (PMC7417307; doi:10.3389/fbioe.2020.00798)
Supplement: Supplementary file 1 [file Data_Sheet_1.PDF]

Supplementary Material of ”Identifying the potential mechanism of action of SNPs associated with breast cancer susceptibility with GVITamIN”

An-phi Nguyen[1,2,*], Paola Nicoletti[3,4,*], Damien Arnol[1,5], Andrea Califano[3] and María Rodríguez Martínez[3, 6 †]
[1] IBM Research – Zürich, Switzerland.
[2] ETH - Zürich, Switzerland.
[3] Columbia University Medical Center, Herbert Irving Cancer Research Center, NY, USA.
[4] Presently at Icahn School of Medicine at Mount Sinai, NY, USA.
[5] Presently at J2-Reliance Ltd, London, E11EQ, United-Kingdom.
[6] Presently at IBM Research – Zürich.
[*] Equally contributing.
[†] Corresponding authors: *mrm@zurich.ibm.com* & *ac2248@cumc.columbia.edu*.

1 COMMENTS ON THE CHOICE OF STATISTICAL TESTS

1.1 First-order analysis

Detecting differential gene expression: As presented in the main paper, for the first-order analysis, we opt for the *Mann-Whitney U-test* [1] to find SNPs that associate with the differential expression of some genes. The test is performed by computing a statistic U, which depends on the number and ranks of the gene expressions of the samples in each group. Note that the distribution of U under the null hypothesis is known and easy to tabulate if the sizes of the two samples are small enough. However, for big sample sizes, greater than $\sim$ 20-30, the distribution can be approximated by a normal distribution.

This non-parametric test has important advantages compared to the commonly used *Student's t-test*. First, as all nonparametric tests, there is no need to make particularly strong assumptions about the data distributions, resulting in increased robustness against outliers, noisy measurements, or non-normality of the data. Moreover, even if groups are normally distributed, the Mann-Whitney U-test has a relatively high (asymptotic) efficiency when compared to the t-test performed on normally distributed data. This means that, in this case, the Mann-Whitney U-test would need just a few more observations than the t-test to achieve asymptotically the same statistical power.

2 ASYMPTOTIC CORRELATION OF CORRELATIONS

Overview of the computation of global SNP-TF association p-values: Given two populations A and B, we perform some measurements of some quantities of interest, e.g. gene expression levels. For each population, we obtain a table of measurements denoted as $\mathbf{X}_A \in \mathbb{R}^{M \times N_A}$ and $\mathbf{X}_B \in \mathbb{R}^{M \times N_B}$, where M is the number of measured quantities, e.g. the number of genes, and N_A and N_B are the samples sizes. Let us consider a particular quantity x_A^k, x_B^k with $k \in \{1, \ldots, M\}$, e.g. a transcription factor. We are interested in analyzing if its correlation with the other quantities changes between the two populations. To test this, we go through the following steps.

1. In each population, we compute the correlation between the quantity k and the all other quantities i, obtaining a vector of correlations for each population:

$$\boldsymbol{\rho}_A = \begin{bmatrix} \vdots \\ \rho_A^i \\ \vdots \end{bmatrix} \quad \boldsymbol{\rho}_B = \begin{bmatrix} \vdots \\ \rho_B^i \\ \vdots \end{bmatrix} ,$$

   where $\rho_A^i = \rho(x_A^k, x_A^i)$, where $\rho(x,y) = \frac{\mathrm{Cov}(x,y)}{\sigma_x \sigma_y}$ is Pearson correlation and $i = 1, \ldots, M$ is the index over the measured quantities.

2. We now transform the correlations using Fisher's z-transform, obtaining the following two vectors:

$$\mathbf{z}_A = \begin{bmatrix} \vdots \\ z_A^i \\ \vdots \end{bmatrix} \quad \mathbf{z}_B = \begin{bmatrix} \vdots \\ z_B^i \\ \vdots \end{bmatrix} ,$$

   where $z_A^i = \operatorname{arctanh}(\rho_A^i)$ (Eq. 1 in the main text).

3. The transformed variables obtained in the previous step are normally distributed with standard deviations approximately equal to $\frac{1}{\sqrt{N_A - 3}}$ and $\frac{1}{\sqrt{N_B - 3}}$ respectively. Hence, we can use the *z-test* to test whether the correlation between quantities k and i is statistically different in populations A and B. Under the null hypothesis, we have that

$$t_i = \frac{z_A^i - z_B^i}{\sqrt{\frac{1}{N_A - 3} + \frac{1}{N_B - 3}}} \sim \mathcal{N}(0,1) \; \forall i \,, \tag{S1}$$

   where $\mathcal{N}(0,1)$ is the standard normal distribution. Finally from Equation S1, we obtain a vector of p-values:

$$\mathbf{p} = \begin{bmatrix} \vdots \\ p^i \\ \vdots , \end{bmatrix}$$

   where p^i is the p-value obtained by comparing z_A^i and z_B^i as described above.

4. In the last step of our analysis, we combine all the p-values to reach a global conclusion about the quantity k. In a biological context, for example, this could allow us to answer the question: Is the activity of transcription factor k disrupted in one population but not in the other? To do so, we apply *Fisher's combination method*

$$P = -2 \sum_{i=1}^{M} p^i .$$

   If the tests were independent, then P would follow a chi-square distribution with $2M$ degrees of freedom, i.e. $P \sim \chi^2_{2M}$. In case of dependence, P follows a differently scaled chi-square distribution. M. Brown [2] found an approximation of the rescaling factors that is dependent on the correlations between the statistics computed in Eq. S1, $r_{ij} = \rho(t_i, t_j)$.

The problem: As explained in the previous section, to account for data dependencies using Brown method, we have to compute a rescaling factor that depends on the correlations r_{ij}. However, after applying GVITamIN pipeline, we are left with only single point estimate for each t_i and, hence, we cannot compute the correlation by just applying Pearson formula. The brute force solution is to create various bootstrap samples and apply GVITamIN analysis several times to obtain several estimates for each t_i, from which a Pearson correlation can be computed. However, such an approach can be very computationally intensive. In the next section, we introduce an alternative approach that does not require massive computation, relying instead on computing an analytical asymptotic approximation of the correlations r_{ij}.

Derivation of an analytical asymptotic approximation of the correlations

Computing the covariance of Fisher's z-transformed correlations: To simplify the notation, we focus first on only one population and drop therefore the population subscripts. Furthermore, we can focus only on two quantities, apart from the quantity k with respect to which we want to compute correlation. For instance, we can focus only on the transcription factor and two target genes. The reasoning can be trivially extended to more quantities. In the following:

- X is the reference quantity k, e.g. the transcription factor;
- Y and Z are other two measured quantities, e.g. two target genes.

We assume to have a sample of N i.i.d. vectors $(X_1, Y_1, Z_1), \ldots, (X_i, Y_i, Z_i), \ldots, (X_N, Y_N, Z_N)$. Without loss of generality, we can further assume that $\mathbb{E}[X_i] = \mathbb{E}[Y_i] = \mathbb{E}[Z_i] = 0$. We also assume that these variables have *bounded* moments up to the fourth order. We now define the random vector

$$\mathbf{m} = \begin{bmatrix} m_x \\ m_y \\ m_z \\ m_{xy} \\ m_{xz} \\ m_{x^2} \\ m_{y^2} \\ m_{z^2} \end{bmatrix} = \frac{1}{N} \begin{bmatrix} \sum_i X_i \\ \sum_i Y_i \\ \sum_i Z_i \\ \sum_i X_i Y_i \\ \sum_i X_i Z_i \\ \sum_i X_i^2 \\ \sum_i Y_i^2 \\ \sum_i Z_i^2 \end{bmatrix} \in \mathbb{R}^8$$

Thanks to the *Central Limit Theorem*, we have that for $N \to \infty$:

$$\sqrt{N} \left(\mathbf{m} - \begin{bmatrix} 0 \\ 0 \\ 0 \\ \sigma_{xy} \\ \sigma_{xz} \\ \sigma_x^2 \\ \sigma_y^2 \\ \sigma_z^2 \end{bmatrix} \right) \to \mathcal{N}(0, \Sigma) \,,$$

where $\mathcal{N}(0, \Sigma)$ is the multivariate normal distribution with zero mean and covariance matrix

$$\Sigma = \begin{bmatrix} \text{Cov}(X_i, X_i) & & & \cdots & \text{Cov}(X_i, Z_i^2) \\ \text{Cov}(Y_i, X_i) & \text{Cov}(Y_i, Y_i) & & \cdots & \text{Cov}(Y_i, Z_i^2) \\ \text{Cov}(Z_i, X_i) & \text{Cov}(Z_i, Y_i) & \text{Cov}(Z_i, Z_i) & \cdots & \text{Cov}(Z_i, Z_i^2) \\ \text{Cov}(X_iY_i, X_i) & & & \cdots & \text{Cov}(X_iY_i, Z_i^2) \\ \text{Cov}(X_iZ_i, X_i) & & & \cdots & \text{Cov}(X_iZ_i, Z_i^2) \\ \text{Cov}(X_i^2, X_i) & & & \cdots & \text{Cov}(X_i^2, Z_i^2) \\ \text{Cov}(Y_i^2, X_i) & & & \cdots & \text{Cov}(Y_i^2, Z_i^2) \\ \text{Cov}(Z_i^2, X_i) & & & \cdots & \text{Cov}(Z_i^2, Z_i^2) \end{bmatrix} \in \mathbb{R}^{8\times 8}$$

To compute the correlations between the t_i as explained in the previous section, our first step is to find the covariance between the (estimates of the) correlations of the original quantities:

$$\rho_y = \rho(X, Y) = \frac{s_{xy}}{s_x s_y} \quad \rho_z = \rho(X, Z) = \frac{s_{xz}}{s_x s_z}\,,$$

with

$$s_x^2 = m_{x^2} - m_x^2, \quad s_y^2 = m_{y^2} - m_y^2, \quad s_z^2 = m_{z^2} - m_z^2$$
$$s_{xy} = m_{xy} - m_x m_y, \quad s_{xz} = m_{xz} - m_x m_z\,.$$

As a first step, we define the mapping $g : \mathbb{R}^8 \mapsto \mathbb{R}^5$:

$$g(\mathbf{m}) = \begin{bmatrix} s_x^2 \\ s_y^2 \\ s_z^2 \\ s_{xy} \\ s_{xz} \end{bmatrix} = \begin{bmatrix} m_{x^2} - m_x^2 \\ m_{y^2} - m_y^2 \\ m_{z^2} - m_z^2 \\ m_{xy} - m_x m_y \\ m_{xz} - m_x m_z \end{bmatrix}.$$

We can then apply the *Delta method* [3] and find that:

$$\sqrt{N}\left(\begin{bmatrix} s_x^2 \\ s_y^2 \\ s_z^2 \\ s_{xy} \\ s_{xz} \end{bmatrix} - \begin{bmatrix} \sigma_x^2 \\ \sigma_y^2 \\ \sigma_z^2 \\ \sigma_{xy} \\ \sigma_{xz} \end{bmatrix} \right) \to \mathcal{N}(0, \Sigma^*)\,,$$

where $\Sigma^* = \dot{g}(\mathbf{m})\Sigma\dot{g}(\mathbf{m})^T$ is the Jacobian of g:

$$\dot{g}(\mathbf{m}) = \begin{bmatrix} -2m_x & 0 & 0 & 0 & 0 & 1 & 0 & 0 \\ 0 & -2m_y & 0 & 0 & 0 & 0 & 1 & 0 \\ 0 & 0 & -2m_z & 0 & 0 & 0 & 0 & 1 \\ -m_y & -m_x & 0 & 1 & 0 & 0 & 0 & 0 \\ -m_z & 0 & -m_x & 0 & 1 & 0 & 0 & 0 \end{bmatrix}.$$

By defining $h : \mathbb{R}^5 \mapsto \mathbb{R}^2$ as:

$$h(\begin{bmatrix} s_x^2 \\ s_y^2 \\ s_z^2 \\ s_{xy} \\ s_{xz} \end{bmatrix}) = \begin{bmatrix} \rho_y \\ \rho_z \end{bmatrix} = \begin{bmatrix} \frac{s_{xy}}{\sqrt{(s_x^2 s_y^2)}} \\ \frac{s_{xz}}{\sqrt{(s_x^2 s_z^2)}} \end{bmatrix} ,$$

we can apply the Delta Method again, and obtain:

$$\sqrt{N}\left(\begin{bmatrix} \rho_y \\ \rho_z \end{bmatrix} - \begin{bmatrix} \mu_{\rho_y} \\ \mu_{\rho_z} \end{bmatrix} \right) \to \mathcal{N}(0, \tilde{\Sigma}) ,$$

where $\tilde{\Sigma} = \dot{h}(\mathbf{s})\Sigma^* \dot{h}(\mathbf{s})^T$ and $\dot{h}(\mathbf{s})$ is the Jacobian of h:

$$\dot{h}(\mathbf{s}) = \begin{bmatrix} -\frac{s_{xy}}{2s_x^3 s_y} & -\frac{s_{xy}}{2s_x s_y^3} & 0 & \frac{1}{s_x s_y} & 0 \\ -\frac{s_{xz}}{2s_x^3 s_z} & 0 & -\frac{s_{xz}}{2s_x s_z^3} & 0 & \frac{1}{s_x s_z} \end{bmatrix} .$$

Finally, we apply Fisher's z-transform $F : \mathbb{R}^2 \mapsto \mathbb{R}^2$ to obtain an expression for z_y and z_z as follows:

$$F(\begin{bmatrix} \rho_y \\ \rho_z \end{bmatrix}) = \begin{bmatrix} z_y \\ z_z \end{bmatrix} = \begin{bmatrix} \operatorname{arctanh}(\rho_y) \\ \operatorname{arctanh}(\rho_z) \end{bmatrix} .$$

The covariance for the z-transformed correlations is then $\hat{\Sigma} = \dot{F}(\boldsymbol{\rho})\tilde{\Sigma}\dot{F}(\boldsymbol{\rho})^T$ with:

$$\dot{F}(\boldsymbol{\rho}) = \begin{bmatrix} \frac{1}{1-\rho_y^2} & 0 \\ 0 & \frac{1}{1-\rho_z^2} \end{bmatrix} .$$

Computing the correlation between t_i: We are now ready to compute the correlation that we need. From the previous subsection, we know that the Fisher z-transformed correlations are (asymptotically) distributed as:

$$\frac{\mathbf{z}_A - \boldsymbol{\mu}_A}{\sqrt{\frac{1}{N_A-3}}} \sim \mathcal{N}(0, \hat{\Sigma}_A) ,$$

and

$$\frac{\mathbf{z}_B - \boldsymbol{\mu}_B}{\sqrt{\frac{1}{N_B-3}}} \sim \mathcal{N}(0, \hat{\Sigma}_B) .$$

Equivalently:

$$\mathbf{z}_A - \boldsymbol{\mu}_A \sim \mathcal{N}(0, \frac{\hat{\Sigma}_A}{N_A - 3}) ,$$

and

$$\mathbf{z}_B - \boldsymbol{\mu}_B \sim \mathcal{N}(0, \frac{\hat{\Sigma}_B}{N_B - 3}) .$$

We know from the first section that the t_i follow a normal distribution, i.e.:

$$\frac{\mathbf{t} - \boldsymbol{\mu}_t}{\sqrt{\frac{1}{N_A-3} + \frac{1}{N_B-3}}} \sim \mathcal{N}(0, \bar{\Sigma}),$$

or, equivalently,

$$\mathbf{t} - \boldsymbol{\mu}_t \sim \mathcal{N}(0, \left(\frac{1}{N_A - 3} + \frac{1}{N_B - 3}\right)\bar{\Sigma}).$$

Furthermore, as the covariance of the sum of normally distributed variables is the sum of the covariances of the variables, we can equate the covariances and obtain:

$$\left(\frac{1}{N_A - 3} + \frac{1}{N_B - 3}\right)\bar{\Sigma} = \frac{\hat{\Sigma}_A}{N_A - 3} + \frac{\hat{\Sigma}_B}{N_B - 3},$$

and

$$\bar{\Sigma} = \frac{(N_B - 3)\hat{\Sigma}_A + (N_A - 3)\hat{\Sigma}_B}{N_A + N_B - 6}.$$

We finally arrive to an expression between the correlation between t_i and t_j, as:

$$\rho(t_i, t_j) = \frac{\bar{\Sigma}_{12}}{\sqrt{\bar{\Sigma}_{11}\bar{\Sigma}_{22}}}.$$

How to apply this? In practice, we just need to compute the initial correlations $\boldsymbol{\rho}$ and the covariance matrix of the moments $\mathbf{m}$. We can then apply all the transformations to the covariance matrix as described above and finally obtain the needed correlations of t_i.

What about Spearman's correlation: Given a fixed sample size N, the $rg(X_i)$ (where $rg(\cdot)$ is the function that transforms the sample points to their ranking in the population) are i.i.d. variables. Since the Spearman's correlation is just the Pearson's correlation applied to the rank variables, we just need to first apply the transformation $\tilde{X}_i = rg(X_i)$ and then we can apply verbatim the same reasoning as above.

3 LIST OF SNPS USED FOR THE ANALYSIS

The goal of GVITamIN is to unveil possible molecular mechanisms behind SNPs associated with complex diseases such as breast cancer, and hence, we focus on the analysis of 59 SNPs already known to increase the risk of breast cancer. The list is obtained by overlapping the SNPs mentioned on SNPedia (`https://www.snpedia.com/index.php/Breast_cancer`) with the SNPs for which the data was available from TCGA. The SNPs finally considered in this paper are:

rs421379	rs614367	rs704010	rs737387	rs921551	rs1078806
rs1092913	rs1219648	rs1292011	rs1314913	rs1562430	rs1800056
rs1801270	rs1876206	rs1926657	rs1978503	rs2046210	rs2048672
rs2056116	rs2075555	rs2180341	rs2380205	rs2386661	rs2774307
rs2842347	rs2854344	rs2981575	rs2981582	rs3784099	rs3785982
rs3803662	rs3817198	rs3884558	rs4322600	rs4455437	rs4784227
rs4987047	rs6556756	rs7107217	rs9383951	rs9485372	rs9628987

rs9934948	rs10411161	rs10490113	rs10509373	rs10510102	rs10822013
rs10853029	rs10871290	rs11613298	rs12255372	rs12711517	rs12762549
rs13393577	rs16882214	rs17051310	rs17141741	rs17435444	

4 ADDITIONAL FIGURES

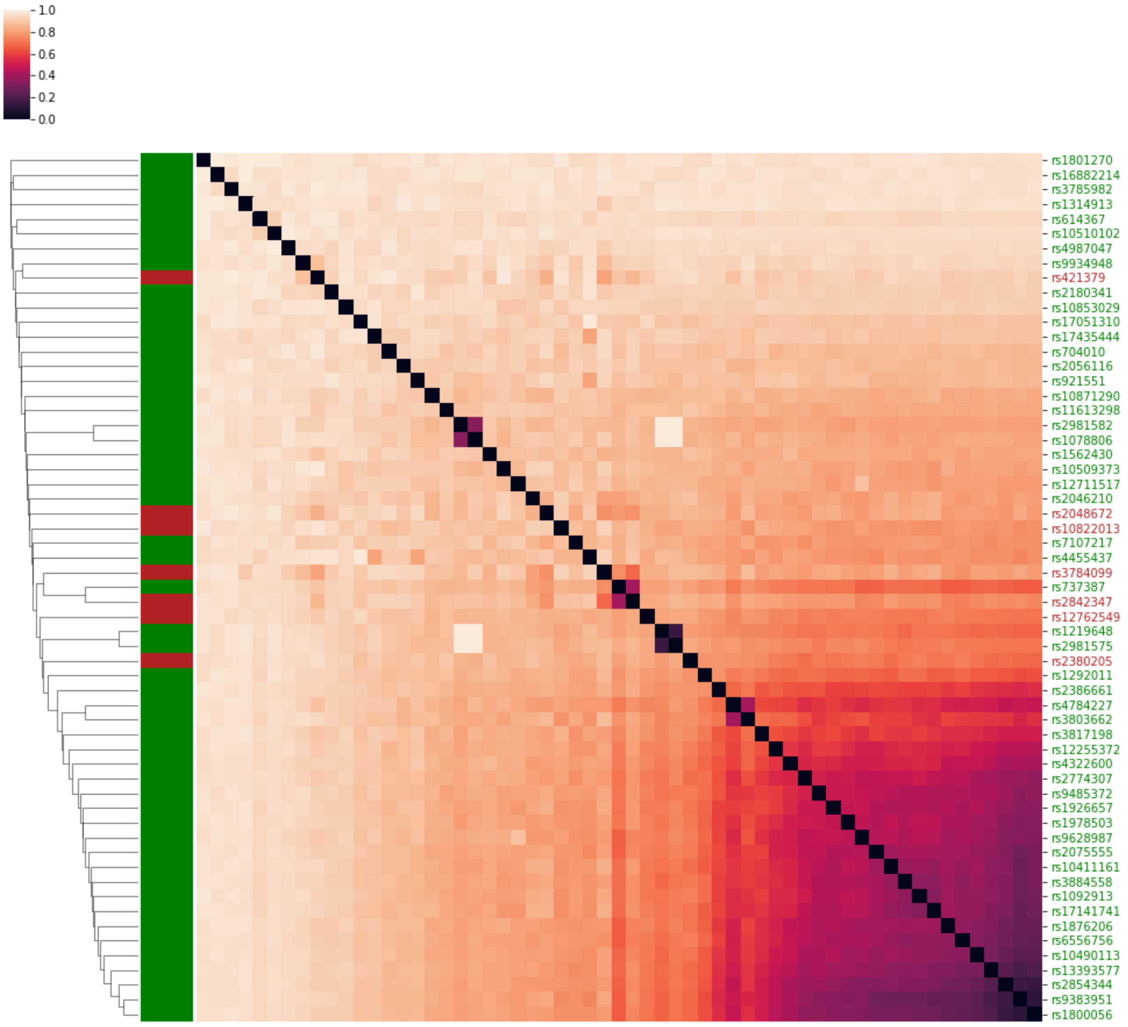

Figure S1. Clustering of SNPs according to the jaccard index of the cohort splitting they induce. In red are highlighted the SNPs which are found correlated to LEF1. If two SNPs belong to the same cluster it means that they produce similar cohort splitting. The SNPs correlated to LEF1 do not form a clear cluster, hence providing evidence that LEF1 may play a central role in cancer.

REFERENCES

[1]H. B. Mann and D. R. Whitney. On a test of whether one of two random variables is stochastically larger than the other. *Ann. Math. Statist.*, 18(1):50–60, 03 1947.

[2]Morton B. Brown. 400: A method for combining non-independent, one-sided tests of significance. *Biometrics*, 31(4):987–992, 1975.

[3]C. Cox. *Delta Method*. American Cancer Society, 2005.
